# A powered simple walking model explains the decline in propulsive force and hip flexion torque compensation in human gait

**DOI:** 10.1038/s41598-023-41706-0

**Published:** 2023-09-07

**Authors:** Hajime Ohtsu, Kazunori Hase, Kouta Sakoda, Shinya Aoi, Shunsuke Kita, Shinya Ogaya

**Affiliations:** 1 Department of Mechanical Science and Bioengineering, Graduate School of Engineering Science, Osaka University, 1-3 Machikaneyama, Toyonaka, Osaka 560-8531 Japan; 2 Japan Society for the Promotion of Science, Tokyo, Japan; 3 Department of Mechanical Systems Engineering, Faculty of Systems Design, Tokyo Metropolitan University, Tokyo, Japan; 4 Department of Mechanical Systems Engineering, Graduate School of Systems Design, Tokyo Metropolitan University, Tokyo, Japan; 5 Department of Health and Social Services, Graduate School of Saitama Prefectural University, Saitama, Japan; 6 Department of Physical Therapy, Touto Rehabilitation College, Tokyo, Japan; 7 Department of Rehabilitation, Soka Orthopedics Internal Medicine, Saitama, Japan; 8 Department of Physical Therapy, Saitama Prefectural University, Saitama, Japan

**Keywords:** Computational models, Biomechanics, Differential equations, Numerical simulations, Geriatrics, Mechanical engineering

## Abstract

Excessive hip flexion torque to prioritize leg swings in the elderly is likely to be a factor that reduces their propulsive force and gait stability, but the mechanism is not clear. To understand the mechanism, we investigated how propulsive force, hip flexion torque, and margin of stability (MoS) change when only the hip spring stiffness is increased without changing the walking speed in the simple walking model, and verified whether the relationship holds in human walking. The results showed that at walking speeds between 0.50 and 1.75 m/s, increasing hip spring stiffness increased hip flexion torque and decreased the propulsive force and MoS in both the model and human walking. Furthermore, it was found that the increase in hip flexion torque was explained by the increase in spring stiffness, and the decreases in the propulsive force and MoS were explained by the increase in step frequency associated with the increase in spring stiffness. Therefore, the increase in hip flexion torque likely decreased the propulsive force and MoS, and this mechanism was explained by the intervening hip spring stiffness. Our findings may help in the control design of walking assistance devices, and in improving our understanding of elderly walking strategies.

## Introduction

Walking speed is modulated by the propulsive force (i.e., the anterior component of the ground reaction force) generated during the push-off phase, and both the comfortable walking speed and propulsive force decrease significantly with age^1, 2^. A walking speed of less than 1.0 m/s in elderly adults is a strong predictor of falling down while walking^3^, and preventing their falls would require an enhanced propulsive force to maintain their walking speed. However, even if older adults walk at the same speed as younger adults, they generate smaller propulsive forces during the push-off phase^2^. Underlying this reduced propulsive force is a neuromuscular compensatory mechanism that offsets the age-related decrease in plantar flexor power generation by an increase in proximal muscle mobilization^4^. Specifically, this compensation is an increase in hip flexion torque to compensate for the decrease in ankle plantar flexion torque during the push-off phase^5^. In other words, elderly adults prioritize leg swings using hip flexors over propulsion using ankle plantar flexors. Therefore, although their excessive increase in hip flexion torque to prioritize leg swing is likely a contributing factor to reduced propulsive force and gait stability, the mechanism is not clear.

Parametric studies using walking models are useful for elucidating such mechanisms. By defining propulsive force, hip flexion torque, and gait stability as variables, we can understand their interrelationships. Thus, we focused on the simplest walking model^6^ with torque springs between both legs. This hip spring well mimics the muscle activity of the proximal muscles of the lower limb. In human gait, the hip flexion torque just before toe-off accelerates the leg for swinging, while the hip extension torque and knee flexion torque in the terminal swing decelerate the swing leg in preparation for heel contact^7^. The hip spring also generates the hip flexion torque in the initial swing and the hip extension torque in the terminal swing, and these torques increase as the spring is stiffened. Therefore, we attempted to reproduce hip muscle activity by spring stiffness and to calculate hip flexion torque. In addition, as in our previous study^8^, the impulse generated by the push-off (push-off impulse) was applied to the model during the double support period to calculate the propulsive force. Here, the double support period is a function of the angular velocity of the stance leg, and the propulsive force is the push-off impulse divided by the period.

In addition, we used the margin of stability (MoS) proposed by Hof et al.^9^ as a measure of gait stability. This is because the MoS is often used as a measure of stability in human gait and to identify older adults with a fall risk^10, 11^. The MoS is determined by the distance between the center of mass including the velocity factor, that is, the extrapolated center of mass (XcoM) and the base of the support boundary^9^. Furthermore, it was reported that the sagittal MoS at heel contact in the elderly is lower than that in the young under the same velocity conditions^10^. Thus, while an increase in hip flexion torque is likely to be associated with a decrease in the MoS, the relationship is not clear. Based on the above, we calculated the MoS from the step length and walking speed of the model.

The purpose of this study was to clarify by what mechanism an increase in hip flexion torque reduces the propulsive force and gait stability. To achieve this purpose, we first investigated how an increase in hip spring stiffness affects hip flexion torque, propulsive force, and the MoS using the simple walking model. Next, we verified the validity of the revealed relationship by human gait experiments.

## Methods

### Equations of motion for the walking model

**Figure 1:**
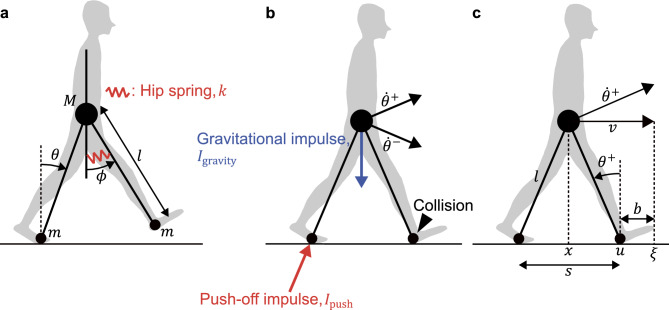
The powered simple walking model. (**a**) A hip spring is attached between the trunk, which is always perpendicular to the ground, and the swing leg. (**b**) Two impulses ($I_\text{push}$, $I_\text{gravity}$) are applied to the model just before the swing leg collides with the ground. (**c**) Post-collision state of the model $$\begin{bmatrix} \theta^+ & \dot{\theta}^+ \end{bmatrix}^T$$ and geometric relationship between step length $s$, walking speed $v$, and margin of stability $b$. The figure shows the geometric relationship when $l=1$.

We used a simple walking model with a torque spring at the hip joint (Fig. 1a). The model has two massless legs of length $l$ (swing and stance legs) connected at the frictionless hip joint and a massless trunk that is assumed to be always perpendicular to the ground. In addition, it has a point mass $M$ at the hip and a point mass $m$ at each foot. The kinematics of this model are described by the stance leg angle $\theta$ relative to the vertical and the swing leg angle $\phi$ relative to the trunk. By setting $\theta$ to positive clockwise and $\phi$ to positive counterclockwise, the direction of those angles was matched with that of the travel direction of the model. In addition, we added a torque spring to the hip of this model, which has a spring constant $k$, similar to the extension of Kuo^6^. This hip spring mimics bursts of hip muscle activity. Therefore, the equations of motion for the model are given by

1 $$\begin{array}{*{20}c} {\left[ {\begin{array}{*{20}c} {1 + \beta } & {\beta \cos \left( {\theta + \phi } \right)} \\ {\beta \cos \left( {\theta + \phi } \right)} & \beta \\ \end{array} } \right]\left[ {\begin{array}{*{20}c} {\mathop \theta \limits^{ \cdot \cdot } } \\ {\mathop \phi \limits^{ \cdot \cdot } } \\ \end{array} } \right] + \left[ {\begin{array}{*{20}c} { - \beta \sin \left( {\theta + \phi } \right)\dot{\phi }^{2} } \\ { - \beta \sin \left( {\theta + \phi } \right)\dot{\theta }^{2} } \\ \end{array} } \right] + \left[ {\begin{array}{*{20}c} { - \left( {1 + \beta } \right)\sin \theta } \\ {\beta \sin \phi } \\ \end{array} } \right] = \left[ {\begin{array}{*{20}c} 0 \\ { - k\phi } \\ \end{array} } \right]} \\ \end{array}$$

where $\beta =m/M=0.074$. $M$ and $m$ were determined by referring to the upper and lower body masses, respectively, from anthropometric data^7^. Note that $m$ was determined such that the moment of inertia $m{l}^{2}$ around the hip joint is equal to the moment of inertia of the lower limb around the hip joint in humans. The first term on the left-hand side of Eq. (1) is the inertia term, the second is the Coriolis force and centrifugal force term, and the third is the gravity term, and the right-hand side is the external force term. Equation (1) is expressed in dimensionless terms following basic units: overall mass $M$, leg length $l$, and time $\sqrt{l/g}$, where $g$ is the gravitational acceleration. Note that subsequent equations are similarly expressed in dimensionless terms. Now we linearize the given equation of motion. We assume micro-oscillations of $\theta \ll 1$, $\phi \ll 1$, $\theta +\phi \ll 1$, $\dot{\theta }\ll 1$, and $\dot{\phi }\ll 1$. Hence, because $\sin \theta \approx \theta$, $\cos \theta \approx 1$, $\sin(\theta+\phi) \approx \theta+\phi$, $\cos(\theta+\phi) \approx 1$, $\dot{\theta}^2 \approx 0$, and $\dot{\phi}^2 \approx 0$, the equations of motion are linearized as follows:

2 $$\ddot{\theta}+\dfrac{\beta}{1+\beta}\ddot{\phi}-\theta=0$$

3 $$\ddot{\phi}+\ddot{\theta}+\omega^2\phi=0$$

where $\omega \triangleq \sqrt{1+k/\beta }$ is the natural frequency of the swing leg.

### Step-to-step transition rule

Collision of the swing leg with the ground occurs under the following condition:

4 $$\theta - \phi = 0$$

However, when Eq. (1) is integrated numerically, Eq. (4) is first detected in the mid-swing. This is the scuffing of the swing leg on the ground^6, 12, 13^, which we ignore in this study because we do not focus on this phenomenon. Furthermore, because the collision between the foot and the ground is assumed to be instantaneous and perfectly inelastic, the angular momentum of the model is conserved before and after the collision. However, because the mechanical energy is dissipated by the collisions, the model cannot walk periodically on level ground without any energy input. Therefore, two impulses were applied to the model just before the collision of the swing leg (Fig. 1b). First, a push-off impulse $I_\text{push}$ was applied along the trailing leg. The impulse is an actuator proposed by Kuo^6^ that mimics the extension of the ankle and knee joints caused by the muscular activity of the soleus and gastrocnemius just prior to toe-off in human walking. Next, a gravitational impulse $I_\text{gravity}$ was applied in the vertical downward direction. This impulse is expressed as gravity integrated over the double support time, mimicking the effect of gravity on a human during the double support phase^14^. We assumed that this impulse applied only to the point mass $M$. Therefore, the post-collision state can be represented by applying $I_\text{push}$ and $I_\text{gravity}$ to the pre-collision state as follows:

5 $$\begin{bmatrix} \theta^+ \\ \dot{\theta}^+ \\ \phi^+ \\ \dot{\phi}^+ \end{bmatrix} = \begin{bmatrix} -\theta^- \\ \dfrac{\cos2\theta^-}{1+\beta\sin^22\theta^-}\dot{\theta}^- \\ -\theta^- \\ -\dfrac{\cos^22\theta^-}{1+\beta\sin^22\theta^-}\dot{\theta}^- \end{bmatrix} + \begin{bmatrix} 0 \\ \dfrac{\sin2\theta^-}{1+\beta\sin^22\theta^-} \\ 0 \\ -\dfrac{\sin4\theta^-}{2+2\beta\sin^22\theta^-} \end{bmatrix} I_\text{push} + \begin{bmatrix} 0 \\ -\dfrac{\sin\theta^-}{1+\beta\sin^22\theta^-} \\ 0 \\ \dfrac{\sin\theta^-\cos2\theta^-}{1+\beta\sin^22\theta^-} \end{bmatrix} I_\text{gravity}$$

where $*^-$ and $*^+$ are the state $*$ just before and after collision, respectively. Furthermore, similar to our previous study^8^, we determined $I_\text{gravity}$ by assuming that the double support period $\Delta t^\text{DS}$ is inversely proportional to $\dot{\theta }^-$ as follows:

6 $$I_\text{gravity}=\Delta t^\text{DS}=\dfrac{\mu}{\dot{\theta}^-}$$

where $\mu$ is the proportionality constant. We validated the above inverse proportionality assumption by calculating $\mu$ by regression analysis using human gait data, which we describe below.

### Poincaré map

We set the Poincaré section just after the collision (at the start of the step). Because $\phi^+=\theta^+$ and $\dot{\phi}^+=-\dot{\theta}^ + \cos 2\theta^+$ from Eq. (5), $\phi^+$ and $\dot{\phi}^+$ are determined from $\theta^+$ and $\dot{\theta }^+$. Therefore, the Poincaré section is a two-dimensional surface $$\begin{bmatrix} \theta & \dot{\theta} \end{bmatrix}^T$$ in a four-dimensional state space $$\begin{bmatrix} \theta & \dot{\theta} & \phi & \dot{\phi} \end{bmatrix}^T$$. From now on, we focus our analysis on this Poincaré section and define the state $q$ as

7 $$q=\begin{bmatrix} \theta & \dot{\theta} \end{bmatrix}^T$$

We suppose a gait in which there is no work for the point mass $M$ during the single support phase and the energy loss due to the collision is fully compensated by $I_\text{push}$ (i.e., $\dot{\theta}^-=\dot{\theta}^+$). The reason for this assumption is that previous modeling studies^6, 15^ have shown that the energy cost of locomotion is minimized under this condition. This condition, called the optimal push-off hypothesis, is also supported by human walking experiments^14^. If this hypothesis holds, the Poincaré map $f$ corresponds to the $i$th and ($i+1$)th points on the Poincaré section as follows:

8 $$q_{i+1}^+=f(q_i^+,k)$$

where $q_i^+$ is the $i$th post-collision state $q$. The map $f$ is a composite map of the map expressed by the equations of motion (Eq. 1) and boundary condition (Eq. 4) and the map expressed by the jump equation (Eq. 5). In other words, if the optimal push-off hypothesis holds, $q_i^+$ is mapped to $q_{i+1}^+$ if only $k$ is specified. Note that the fixed point $$q^*=\begin{bmatrix} \theta^* & \dot{\theta}^* \end{bmatrix}^T$$ on the Poincaré section satisfies the following relationship:

9 $$q^*=f(q^*,k^*)$$

where $k^*$ is $k$ satisfying $q^*=f(q^*,k)$ and exists uniquely for $q^*$. Therefore, we numerically searched for periodic solutions satisfying Eq. (9) using the Newton–Raphson method.

### Margin of stability, propulsive force, and hip joint torque

In the following equations, the post-collision state $$q^+=\begin{bmatrix} \theta^+ & \dot{\theta}^+ \end{bmatrix}^T$$ is expressed as the step length $s$ and walking speed $v$, respectively, to correspond to the variables describing human walking (Fig. 1c):

10 $$s=-2\sin\theta^+$$

11 $$v=\dot{\theta}^+\cos\theta^+$$

Furthermore, the ranges of $s$ and $v$ were set as follows:

12 $$0<s<1$$

13 $$0<v<1$$

Next, the MoS $b$ at the heel strike in the sagittal plane can be expressed using the XcoM $\xi$ and the anterior boundary of base of support $u$ as follows^9^ (Fig. 1c):

14 $$b = u - \xi$$

Furthermore, assuming for simplicity that the center of mass (CoM) position of the model is the position $x$ of the point mass $M$, $u$ is calculated as

15 $$u = x + \dfrac{s}{2}$$

Because the XcoM is the position of the CoM plus its forward velocity divided by $\sqrt{g/l}$^9^, $\xi$ nondimensionalized by $l$ is calculated as follows:

16 $$\xi = x + v$$

After Eqs. (15) and (16) are substituted into Eq. (14), $b$ can be expressed as

17 $$b = \dfrac{s}{2} - v$$

Thus, the more $s$ decreases with respect to $v$, the more $b$ decreases.

If the optimal push-off hypothesis holds, the mechanical energy $E$ is conserved as follows:

18 $$E_i^+=E_{i+1}^-=E_{i+1}^+=\dfrac{1}{2}(1+\beta\sin^22\theta_i^+)(\dot{\theta}_i^+)^2+\cos\theta_i^++\dfrac{1}{2}k(\theta_i^+)^2$$

where $E_i^+$ and $E_{i+1}^-$ are the mechanical energy of the $i$th post-collision and that of the ($i+1$)th pre-collision, respectively. The first, second, and third terms on the right-hand side of this equation represent the kinetic energy, the potential energy due to the gravity, and the potential energy due to the spring, respectively. Therefore, when the initial condition is the fixed point $$q^*=\begin{bmatrix} \theta^* & \dot{\theta}^* \end{bmatrix}^T$$, the pre- and post-collision conditions satisfy the following relationship from Eq. (18):

19 $$-\theta^-=\theta^+=\theta^*$$

20 $$\dot{\theta}^-=\dot{\theta}^+=\dot{\theta}^*$$

Substituting Eqs. (6), (19), and (20) into the second row of Eq. (5) and solving for $I_\text{push}$, we obtain

21 $$I_\text{push}=-\dot{\theta}^*\tan\theta^*(2\beta\cos^2\theta^*+1)+\dfrac{\mu}{2\dot{\theta}^*\cos\theta^*}=\dfrac{sv(s^2\beta-4\beta-2)}{s^2-4}+\dfrac{\mu}{2v}$$

The propulsive force $F$ generated by push-off is calculated as the forward component of $I_\text{push}$ divided by $\Delta t^\text{DS}$ (Eq. 6) as follows:

22 $$F=\dfrac{I_\text{push}\sin(-\theta^*)}{\Delta t^\text{DS}}=\dfrac{sv}{\mu\sqrt{4-s^2}}I_\text{push}$$

**Figure 2:**
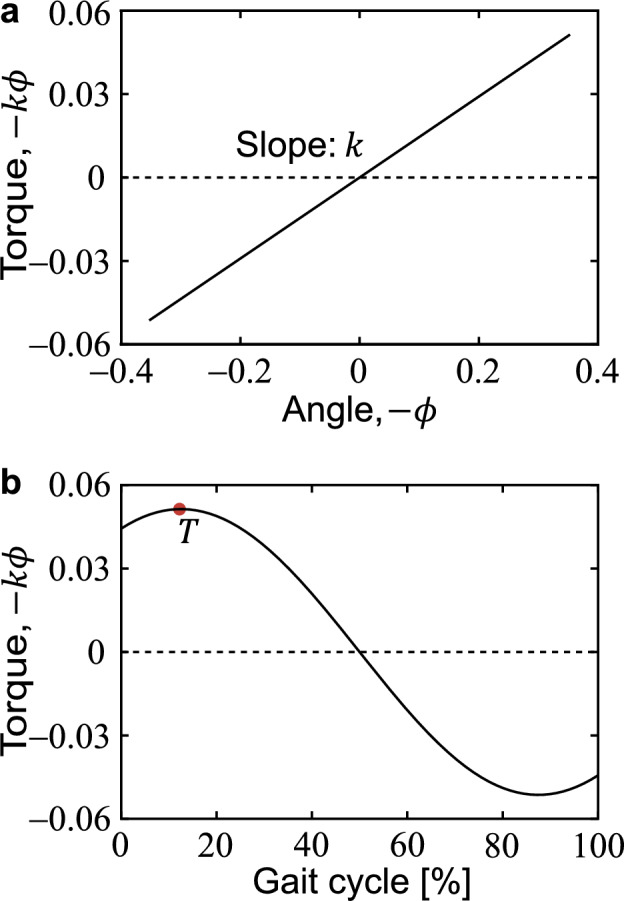
(**a**) Angle $-\phi$ versus torque $-k\phi$ and (**b**) gait cycle versus torque $-k\phi$ when $s=0.6$ and $v=0.39$ are the periodic solution. Positive values of $-\phi$ indicate extension, and positive values of $-k\phi$ indicate flexion torque. The gait cycle is 0% for the post-collision state of one step and 100% for the pre-collision state of the next step.

In addition, the maximum hip flexion torque $T$ can be calculated as the maximum value of the product of the spring constant $k$ and the hip angle $-\phi$ as follows (Fig. 2):

23 $$\begin{array}{*{20}c} {T = \text{max}\left( { - k\phi } \right) = k\phi_{{{\text{max}}}} } \\ \end{array}$$

where $\phi_\text{max}\ge 0$ indicates the hip extension angle when $-k\phi$ takes its maximum value $T$. Next, we obtained the fixed point $q^*$ for any $k$ based on Eq. (9) and then converted it to $s$ and $v$. We also investigated the change of $T$, $F$, and $b$ with increasing $k$ at $v=0.50/\sqrt{g}$, $0.75/\sqrt{g}$, $1.00/\sqrt{g}$, $1.25/\sqrt{g}$, $1.50/\sqrt{g}$, and $1.75/\sqrt{g}$. The six walking speeds $v$ are dimensionless values of the six nominal belt speeds described below.

### Tuning the swing leg

Increasing the hip spring stiffness $k$ leads to shortening the step length $s$ and increasing the walking speed $v$. The rationale is explained below. First, from $\omega \triangleq \sqrt{1+k/\beta }$, increasing $k$ increases the natural frequency $\omega$ of the swing leg. In addition, because the coefficient of $\ddot{\phi }$ in Eq. (2) is $\beta /(1+\beta)\approx 0.192$, the kinematics of the stance leg are not significantly affected by those of the swing leg. Therefore, if the step period is $\tau$, $\omega$ is proportional to the step frequency $1/\tau$:

24 $$\omega \propto \dfrac{1}{\tau}$$

Therefore, increasing $k$ also leads to a shorter step period $\tau$. Because the period $\tau$ is the distance traveled by the CoM divided by its average speed, $\tau$ can be approximated as the step length $s$ divided by the walking speed $v$:

25 $$\tau \approx \dfrac{s}{v}$$

Thus, increasing $k$ leads to shortening the step length $s$ and increasing the walking speed $v$. In addition, Eqs. (24) and (25) were verified by simulation.

### Experimental procedures

The validity of the relationships between the hip spring constant $k$, maximum hip flexion torque $T$, propulsive force $F$, and MoS $b$ in the simulation results was tested by measuring the kinematic and kinetic data of 11 healthy young participants (7 males and 4 females) while walking. When recruiting participants, we excluded those with a history of orthopedic disorders or neurological disorders that may have affected their walking performance. Their average age, height, and mass were 20.5 ± 1.0 years, 1.65 ± 0.10 m, and 58.8 ± 9.1 kg, respectively. This experiment was conducted with the approval of the Ethics Committee of Tokyo Metropolitan University (approval number H3-97) and that of Saitama Prefectural University (approval number 30530). We explained the content of this study to the participants verbally and in writing prior to the experiment and obtained their written informed consent. All experiments were performed in accordance with the Declaration of Helsinki. A three-dimensional motion analysis system (Vicon Nexus, Vicon Motion Systems, Oxford, UK) and a treadmill equipped with two force plates (ITR5018-11, Bertec Corp., OH, USA) were used to measure gait. Kinematic and ground reaction force (GRF) data were recorded at 200 Hz and 1000 Hz, respectively. In addition, kinematic and GRF data were filtered using a fourth-order, low-pass, zero-lag Butterworth filter with cut-off frequencies of 6 Hz and 18 Hz, respectively. Thirty-nine reflective markers were attached to each participant’s entire body in accordance with the Plug-in Gait model.

Participants walked on a treadmill at six different belt speeds ranging from 0.50 to 1.75 m/s in 0.25 m/s increments for 1 min each. Note that these six belt speeds are defined as the nominal belt speeds. The belt speed was scaled to individual leg length by multiplying the nominal belt speed by the square root of the individual’s spina malleolar distance^16^. Participants walked continuously for a 6-min trial and speed-switching periods (from one belt speed to the next). Note that the order of the six different belt speeds was randomized. Before the gait data were measured, the participants walked for several minutes to familiarize themselves with treadmill walking. The participants walked along the X-axis of the laboratory coordinate system. With respect to the traveling direction, the upward direction is the positive direction on the Y-axis.

### Data analysis

The heel strike was defined as the point when the vertical component of the GRF was greater than 0 N, which was detected retrospectively from the point when the GRF was greater than 10 N. Similarly, the toe-off was defined as the point when the vertical component of the GRF was less than 0 N, which was detected prospectively from the point when the GRF was less than 10 N^17^. The heel strike was defined as the starting point of the step, and the next heel strike was defined as the ending point of the step. The last 30 steps of each belt speed were used in the analysis. In the following, we focus on a specific step, belt speed, and participant to derive the variables. Thus, these variables differ depending on the participant, belt speed, and number of steps.

**Figure 3:**
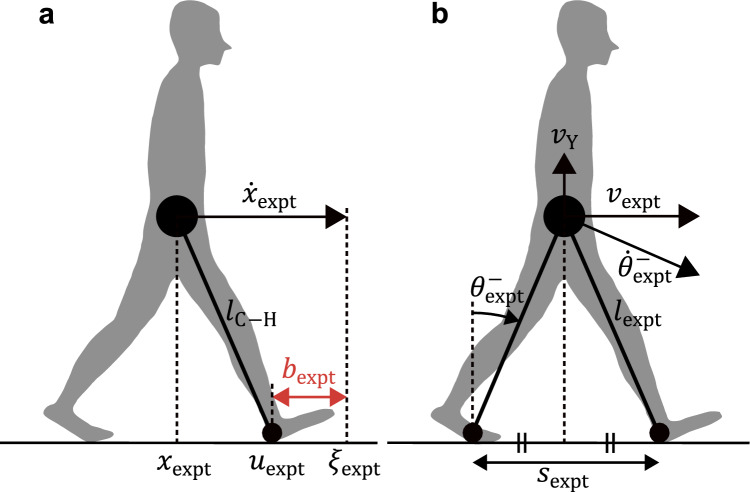
Modeling of human posture at the heel strike. (**a**) Inverted pendulum model for calculating the margin of stability $b_\text{expt}$. The figure shows the geometric relationship when $l_{\text{C}-\text{H}}=1$. (**b**) The posture is represented with the simple walking model. The figure shows the geometric relationship when $l_\text{expt}=1$.

First, as in Eq. (14), the dimensionless MoS at the heel strike $b_\text{expt}$ was calculated as follows (Fig. 3a):

26 $$b_\text{expt}=u_\text{expt}=\xi_\text{expt}$$

where $u_\text{expt}$ is the anterior boundary of the base of support and $\xi_\text{expt}$ is the XcoM at the heel strike. $u_\text{expt}$ is the X coordinate of the heel marker of the leading foot nondimensionalized by $l_{\text{C}-\text{H}}$, where $l_{\text{C}-\text{H}}$ is the distance in the XY coordinates from the CoM to the heel marker of the leading foot. Hereafter, if a variable from the experimental data is shared with a variable $*$ used in the simulation, it is denoted as $*_\text{expt}$. In addition, $\xi_\text{expt}$ was calculated using the position of the CoM at the heel strike in the X coordinate $x_\text{expt}$ and its velocity $\dot{x}_\text{expt}$ as follows:

27 $$\xi_\text{expt} = x_\text{expt} + \dot{x}_\text{expt}$$

where $\dot{x}_\text{expt}$ is the CoM velocity along the X-axis at the heel strike minus the trailing toe marker velocity along the X-axis at the heel strike^18^. This is due to the conversion from treadmill walking to ground walking. In addition, note that $x_\text{expt}$ is nondimensionalized by $l_{\text{C}-\text{H}}$, and $\dot{x}_\text{expt}$ is nondimensionalized by $\sqrt{gl_{\text{C}-\text{H}}}$.

Next, the step length was calculated as the distance on the X-axis between the heel markers of the leading and trailing limb at the heel strike. Here, because step length is a dimensional variable, it must be nondimensionalized using appropriate variables to make comparisons with the simulation results. Therefore, we simplified the human posture at the heel strike to correspond to the walking model. Specifically, we assumed that the vertical projection of the CoM on the ground bisects the step length (Fig. 3b). When this assumption holds, the leg length $l_\text{expt}$ can be calculated from the Pythagorean theorem using the actual CoM height and the actual step length. Hence, the dimensionless step length $s_\text{expt}$ can be calculated by dividing the actual step length by $l_\text{expt}$. Then, the dimensionless walking velocity $v_\text{expt}$ is the CoM velocity along the X-axis at the heel strike minus the trailing toe marker velocity along the X-axis at the heel strike, divided by $\sqrt{gl_\text{expt}}$. Similarly, $v_\text{Y}$ is the CoM velocity along the Y-axis at the heel strike, divided by $\sqrt{gl_\text{expt}}$. In addition, $\theta_\text{expt}^-$ is defined as the angle of the trailing limb relative to the vertical axis. Then, $\dot{\theta}_\text{expt}^-$, the angular velocity of the CoM around the trailing foot at the heel strike, is expressed as follows (Fig. 3b):

28 $$\theta_\text{expt}^-=\sin^{-1}(s_\text{expt}/2)$$

29 $$\dot{\theta}_\text{expt}^-=v_\text{expt}\cos\theta_\text{expt}^--v_\text{Y}\sin\theta_\text{expt}^-$$

Next, to confirm the validity of the prediction of the double support period $\Delta t^\text{DS}$ in the simulation (Eq. 6), we verified whether the double support period $\Delta t_\text{expt}^\text{DS}$ in human gait can be predicted from the angular velocity around the trailing foot at the heel strike $\dot{\theta}_\text{expt}^-$. Here, $\Delta t_\text{expt}^\text{DS}$ was calculated as the period from the heel strike to toe-off divided by $\sqrt{g/l_\text{expt}}$. In addition, a single regression analysis was performed with $\Delta t_\text{expt}^\text{DS}$ as the explanatory variable and $\dot{\theta}_\text{expt}^-$ as the objective variable. The regression equation for inverse proportion (Eq. 6) was used to calculate the proportionality constant $\mu_\text{expt}$ and the coefficient of determination $R^2$.

**Figure 4:**
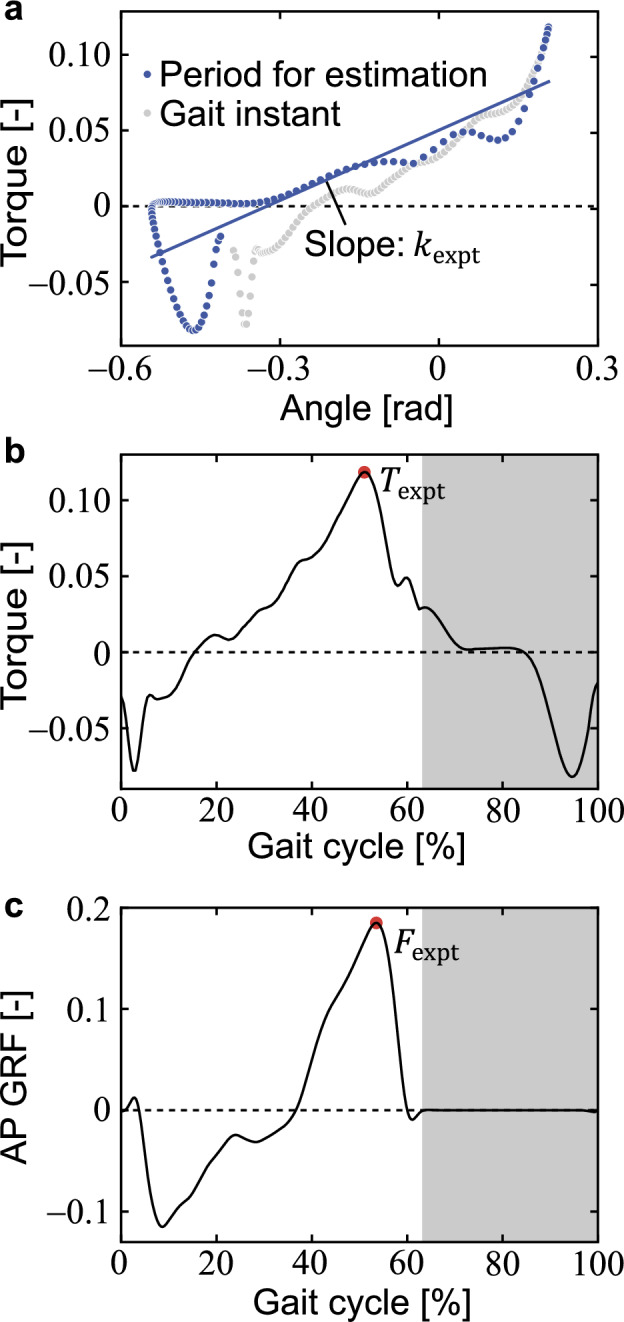
(**a**) Hip joint angle versus hip joint torque, (**b**) gait cycle versus hip joint torque, and (**c**) gait cycle versus anterior–posterior component of the ground reaction force (AP GRF) of the left leg during one gait cycle at a nominal belt speed of 1.25 m/s for one subject. (**a**, **b**) Torque is nondimensionalized by $M_\text{expt}gl_\text{expt}$, whose positive values represent flexion torque, and (**c**) AP GRF is nondimensionalized by $M_\text{expt}g$, whose positive values represent the GRF in the anterior direction. (**a**) Positive hip angles indicate extension. (**b**, **c**) The gait cycle is 0% at the heel contact of the left leg and 100% one frame before the next heel contact of the left leg. White and gray areas indicate the stance and swing phases, respectively.

Next, we estimated the quasi-stiffness $k_\text{expt}$ of the hip joint during the entire swing phase as the slope of a linear fit of the hip joint angle versus torque graph. This quasi-stiffness $k_\text{expt}$ was positioned as the variable corresponding to the torque spring stiffness $k$ in the walking model. It is called “quasi-stiffness” because the stiffness is estimated from the human gait. The quasi-stiffness of the hip joint has been estimated with high accuracy during the hip extension ($R^2=0.92$) and flexion stages ($R^2=0.89$) from the terminal stance to the initial swing phase^19^. This study aimed to estimate the quasi-stiffness that characterizes the effects of the hip joint during the entire swing phase, rather than to create such a strict model. Specifically, we defined the period used for estimation as the period from the maximum hip extension during the pre-swing phase to the end of the swing phase (Fig. 4a), and adopted $k_\text{expt}$ as the slope of a linear fit to the hip angle versus hip joint torque graph during that period. Single regression analysis was used for linear fitting, and a coefficient of determination $R^2$ was obtained for each regression equation.

Finally, the maximum hip flexion torque $T_\text{expt}$ was calculated as the maximum value of the hip joint flexion-extension torque divided by $M_\text{expt}gl_\text{expt}$ (Fig. 4b), where $M_\text{expt}$ is the mass of the participant. The propulsive force $F_\text{expt}$ was calculated as the maximum value of the anterior component (i.e., X-component) of the GRF divided by $M_\text{expt}g$ (Fig. 4c). The step period $\tau_\text{expt}$ was calculated as the period from the contralateral heel strike to the ipsilateral heel strike divided by $\sqrt{l_\text{expt}/g}$.

### Statistical analysis

For the statistical analysis, we defined $$X=\begin{bmatrix} k_\text{expt} \end{bmatrix}$$ and $$Y=\begin{bmatrix} T_\text{expt} & F_\text{expt} & b_\text{expt} & s_\text{expt} \end{bmatrix}$$. To validate whether the relationship between $X$ and $Y$ in human gait is consistent with that in the simulation results, correlation tests were performed between $X$ and $Y$ for each belt speed. Note that each variable is calculated from up to 330 data points (30 steps × 11 persons) for each speed. Pearson’s correlation coefficient was calculated when the two variables were both normally distributed, and Spearman’s rank correlation coefficient was calculated when at least one variable was not normally distributed. Next, a correlation test for $k_\text{expt}$ versus $1/\tau_\text{expt}$ at all speeds was performed to verify whether increasing spring stiffness increases step frequency in human gait as well. The significance level $\alpha$ in this study was set at 0.05. However, since the correlation analysis was repeated four times for one variable $k_\text{expt}$ in the correlation test between $X$ and $Y$, the significance level needed to be adjusted to reduce the type I error. Therefore, the significance level ${\alpha }^{\prime}$ adjusted by the Bonferroni correction^20^ was determined as follows:

30 $$\alpha^{\prime} = 1 - (1-\alpha)^{1/n}$$

where $n$ is the number of iterations of the correlation analysis, which in the case of this study is 4. Thus, the significance level ${\alpha }^{\prime}$ in the correlation test between $X$ and $Y$ was set at 0.01. JASP version 0.16.3 (JASP Team, Amsterdam, Netherlands) was used for all statistical analyses.

## Results

### Simulation results

**Figure 5:**
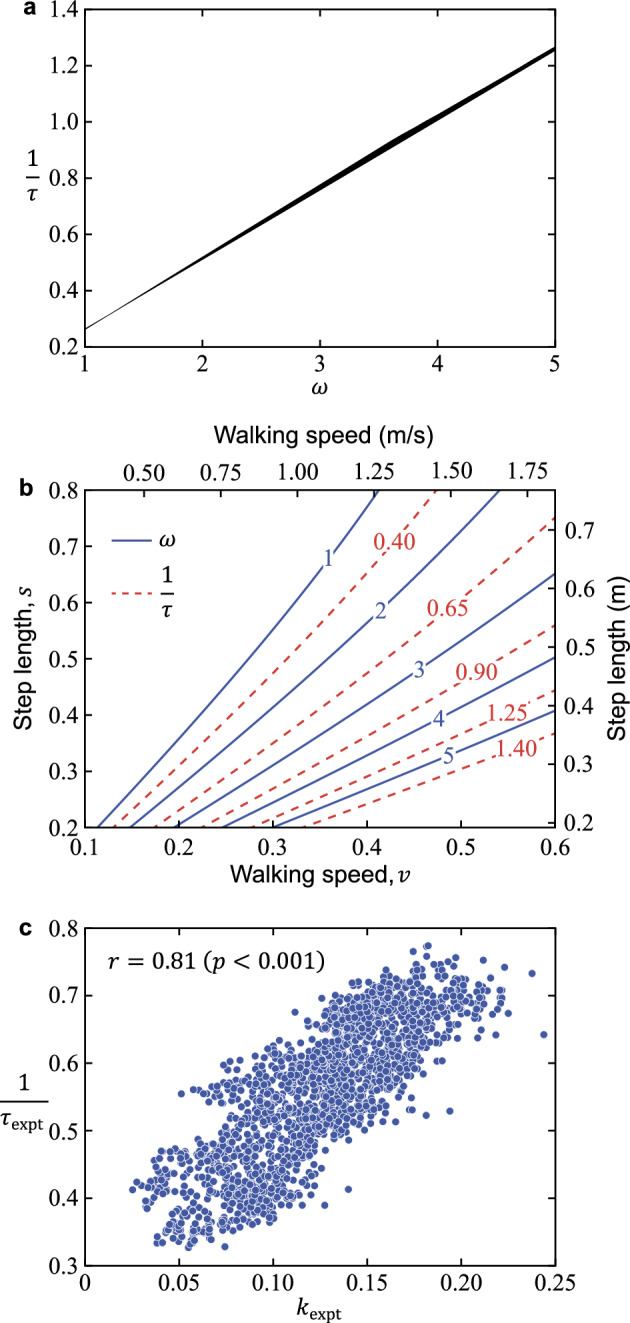
(**a**) Relationship between the natural frequency $\omega$ of the swing leg and step frequency $1/\tau$. The lines show relationships for the entire range of step length ($0<s<1$) and walking speed ($0<v<1$) combinations simulated. (**b**) Contour lines of $\omega$ and $1/\tau$ with respect to walking speed $v$ and step length *s*. $1/\tau =0.40, 0.65, 0.90, 1.25, \mathrm{and}\ 1.40$ correspond to $\tau \approx 2.50, 1.54, 1.11, 0.80, \mathrm{and}\ 0.71$, respectively. The upper and right axes are in SI units for $l=0.96\ \mathrm{m}$ and $g=9.81\ \mathrm{m}/{\mathrm{s}}^{2}$, where $\text {l.}$ is the mean value of $l_\text{expt}$ for all trials (11 people × 6 belt speeds × 30 steps). (**c**) Relationship between the hip quasi-stiffness $k_\text{expt}$ and step frequency $1/\tau_\text{expt}$. The plot shows all data (11 people × 6 belt speeds × 30 steps) in the walking experiment. Values in the figure indicate Spearman’s rank correlation coefficient and *p*-value.

**Figure 6:**
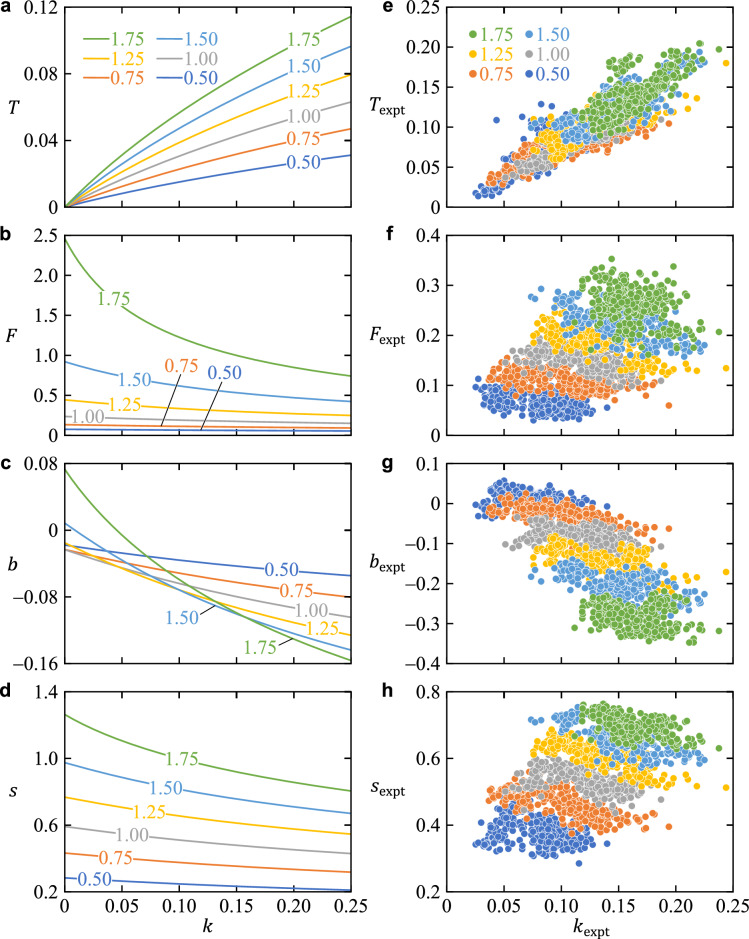
Maximum flexion torque $T$ (**a**, **e**), propulsive force $F$ (**b**, **f**), margin of stability $b$ (**c**, **g**), and step length $s$ (**d**, **h**) with respect to hip spring stiffness $k$ in the simulation and walking experiment. Note that $*_\text{expt}$ indicates that the variable $*$ in the simulation is also used in the walking experiment. The colored contour lines in the simulation results (**a**–**d**) show velocities $v$ ranging from $0.50/\sqrt{g}$ to $1.75/\sqrt{g}$. The colored plots in the experimental results (**e**–**h**) show nominal belt speeds from 0.50 to 1.75 m/s. Note that in (**b**), propulsive force $F$ was calculated using $\mu = 0.142$, where $\mu$ is the mean value of $\mu_\text{expt}$ for all subjects.

The natural frequency $\omega$ of the swing leg was proportional to the step frequency $1/\tau$, as obtained by Eq. (24) (Fig. 5a). Furthermore, the step period $\tau$ was approximated as $s/v$, as obtained by Eq. (25) (Fig. 5b). These indicated that increasing the hip spring constant $k$ leads to shortening the step length $s$ and increasing the walking speed $v$ (Fig. 5b). For all speed conditions of $v=0.50/\sqrt{g}$*, *$0.75/\sqrt{g}$*, *$1.00/\sqrt{g}$*, *$1.25/\sqrt{g}$*, *$1.50/\sqrt{g}$*,* and $1.75/\sqrt{g}$, increasing $k$ increased the maximum hip flexion torque $T$ and decreased the propulsive force $F$, MoS $b$, and step length $s$ (Fig. 6a–d).

### Experimental results

Mean ± sample standard deviation of all subjects for proportionality constant $\mu_\text{expt}$ and coefficient of determination ${R}^{2}$ were 0.142 ± 0.015 and 0.92 ± 0.03, respectively. Therefore, the double support phase $\Delta t_\text{expt}^\text{DS}$ was very well explained from the pre-collision angular velocity $\dot{\theta}_\text{expt}^-$ using the proportionality constant $\mu_\text{expt}$. Because the coefficient of determination $R^2$ of the regression equation for hip joint angle versus torque ranged from 0.68 to 0.79 (Table 1), the quasi-stiffness $k_\text{expt}$ of the hip, the slope of the equation, was well estimated. A significant correlation was found for $k_\text{expt}$ versus step frequency $1/\tau_\text{expt}$ (Fig. 5c). Significant positive correlations were found for $k_\text{expt}$ versus $T_\text{expt}$, and significant negative correlations were found for $k_\text{expt}$ and $F_\text{expt}$, $k_\text{expt}$ and $b_\text{expt}$, and $k_\text{expt}$ and $s_\text{expt}$ for each speed from 0.50 to 1.75 m/s (Fig. 6e–h, Table 2).

**Table 1:** Mean and sample standard deviation of all trials (30 steps × 11 persons) for hip quasi-stiffness $k_\text{expt}$ and coefficient of determination $R^2$ at each speed.

	$k_\text{expt}$	$R^2$
0.50 m/s	0.08 (0.02)	0.72 (0.12)
0.75 m/s	0.10 (0.03)	0.68 (0.14)
1.00 m/s	0.12 (0.03)	0.71 (0.10)
1.25 m/s	0.14 (0.03)	0.72 (0.07)
1.50 m/s	0.15 (0.03)	0.75 (0.07)
1.75 m/s	0.16 (0.02)	0.79 (0.05)

Note that the speeds represent nominal belt speeds, and the actual belt speed is the nominal speed multiplied by the square root of the individual’s spina malleolar distance.

**Table 2:** Correlations between $k_\text{expt}$, the quasi-stiffness of the hip joint during the swing phase, and the variables at each belt speed.

	$T_\text{expt}$	$F_\text{expt}$	$b_\text{expt}$	$s_\text{expt}$
$k_\text{expt}$ at 0.50 m/s	0.72 (< 0.001)*	− 0.46 (< 0.001)*	− 0.36 (< 0.001)*	− 0.26 (< 0.001)*
$k_\text{expt}$ at 0.75 m/s	0.78 (< 0.001)*	− 0.54 (< 0.001)*	− 0.74 (< 0.001)*	− 0.72 (< 0.001)*
$k_\text{expt}$ at 1.00 m/s	0.80 (< 0.001)*	− 0.67 (< 0.001)*	− 0.58 (< 0.001)*	− 0.56 (< 0.001)*
$k_\text{expt}$ at 1.25 m/s	0.80 (< 0.001)*	− 0.60 (< 0.001)*	− 0.54 (< 0.001)*	− 0.77 (< 0.001)*
$k_\text{expt}$ at 1.50 m/s	0.77 (< 0.001)*	− 0.59 (< 0.001)*	− 0.49 (< 0.001)*	− 0.74 (< 0.001)*
$k_\text{expt}$ at 1.75 m/s	0.66 (< 0.001)*	− 0.27 (< 0.001)*	− 0.33 (< 0.001)*	− 0.48 (< 0.001)*

All values represent Spearman’s rank correlation coefficient (*p*-value). Since the adjusted significance level ${\alpha }^{\prime}$ is 0.01, *p* < 0.01 is indicated by * and bold. Note that the speeds represent nominal belt speeds, and the actual belt speed is the nominal speed multiplied by the square root of the individual’s spina malleolar distance.

## Discussion

The simulation results indicate that under a constant walking speed, an increase in the hip spring constant $k$ produced an increase in the maximum hip flexion torque $T$ and a decrease in the propulsive force $F$ and the MoS $b$ (Fig. 6a–c). Furthermore, in human gait at belt speeds from 0.50 to 1.75 m/s, an increase in hip quasi-stiffness $k_\text{expt}$ was significantly associated with an increase in maximum hip flexion torque $T_\text{expt}$ and a decrease in the propulsive force $F_\text{expt}$ and MoS $b_\text{expt}$, similar to the simulation results (Fig. 6e–g, Table 2). The reasons for the above relationships are discussed below, with a focus on the mechanisms involved.

### Effect of spring stiffness on hip flexion torque

In the simulation, the maximum hip flexion torque $T$ increased with increasing hip stiffness $k$ because the rate of decrease in $\phi_\text{max}$ was low relative to the rate of increase in $k$. This is indicated by the fact that in Fig. 6a, $T$ is increasing with increasing $k$ while its slope is decreasing.

For human gait at belt speeds from 0.50 to 1.75 m/s, as in the simulation results, an increase in hip quasi-stiffness $k_\text{expt}$ was significantly associated with an increase in the maximum hip flexion torque $T_\text{expt}$ (Fig. 6e, Table 2). Furthermore, $k_\text{expt}$ was well estimated by the regression equation for hip angle versus torque (Table 1). Therefore, the simulation and experimental results both indicate that the increase in hip flexion torque is explained by an increase in hip spring stiffness.

### Effect of spring stiffness on propulsive force

In the simulation, the reason why an increase in the hip spring constant $k$ decreased the propulsive force $F$ (Fig. 6b) is because an increase in $k$ increases the step frequency $1/\tau$, resulting in a decrease in the step length $s$ (Fig. 6d). First, because the natural frequency $\omega$ of the swing leg is proportional to the step frequency $1/\tau$ (Eq. 24, Fig. 5a), the step length *s* decreased as $k$ increased under the constant walking speed $v$. Next, from Eq. (21), push-off impulse $I_\text{push}$ declines as step length $s$ is decreased. This is because the energy loss due to collision decreases as *s* decreases, and thus less impulse $I_\text{push}$ is needed to compensate for that loss. This decrease in energy loss is because as $|\theta^*|$ decreases (i.e., as $s$ decreases), the coefficient of $\dot{\theta}^-$ in Eq. (5), $\cos 2\theta^-/(1+\beta \sin^2 2\theta^-)$, decreases. Finally, from Eq. (22), $F$ declines as $s$ is decreased. The reason is that the ratio of the horizontal (propulsive) component to the vertical component of $I_{\text{push}}$ is lower because $\sin(-{\theta}^*)$ in Eq. (22) decreases as $|{\theta}^*|$ decreases.

For human gait at belt speeds from 0.50 to 1.75 m/s, as in the simulations, an increase in the hip quasi-stiffness $k_\text{expt}$ was significantly associated with a decrease in propulsive force $F_\text{expt}$ (Fig. 6f, Table 2). The reason for this relationship is probably that an increase in $k_\text{expt}$ is associated with a decrease in step length $s_{\text{expt}}$ under the same speed conditions (Table 2). Reports that a decrease in step length is associated with a decrease in propulsive force during a comfortable gait (1.43 m/s)^21^ and that an increase in trailing limb angle, the angle between the vertical axis and the trailing limb, is associated with an increase in propulsive force^22^ support our results. Furthermore, an increase in $k_\text{expt}$ was significantly associated with an increase in step frequency $1/\tau_\text{expt}$ (Fig. 5c). As in the simulation, this increase in $1/\tau_\text{expt}$ most likely contributes to the decrease in $s_\text{expt}$ at a constant belt speed. Finally, the prediction of the double support period $\Delta t_\text{expt}^\text{DS}$ ($R^2=0.92$) supported the hypothesis that the double support period $\Delta t^\text{DS}$ was inversely proportional to $\dot{\theta}^-$ (Eq. 6) in the simulations.

### Effect of spring stiffness on margin of stability

In the simulation, the reason why an increase in the hip spring constant $k$ decreased the MoS $b$ (Fig. 6c) is also because an increase in $k$ increases the step frequency $1/\tau$, resulting in a decrease in the step length $s$ (Fig. 6d). From Eq. (17), $b$ decreases as $s$ decreases.

For human gait at belt speeds from 0.50 to 1.75 m/s, as in the simulations, an increase in the hip spring constant $k_\text{expt}$ was significantly associated with a decrease in the MoS $b_\text{expt}$ (Fig. 6g, Table 2). This is probably because an increase in $k_\text{expt}$ is associated with a decrease in step length $s_\text{expt}$ under the same speed conditions (Fig. 6h, Table 2). A previous study reported a decrease in the MoS with decreasing step length under comfortable walking conditions^23^. Furthermore, as in the simulation, the decrease in step length $s_\text{expt}$ with increasing $k_\text{expt}$ is probably strongly associated with an increase in $1/\tau_\text{expt}$.

### How increased hip flexion torque reduces propulsive force and MoS

Our simulation results show that increasing the hip spring stiffness increases the hip flexion torque and decreases the propulsive force and MoS. These results were also demonstrated as significant correlations in human gait at belt speeds from 0.50 to 1.75 m/s. Furthermore, as mentioned above, the increase in hip flexion torque is explained by the increase in spring stiffness, and the decrease in the propulsive force and MoS is explained by the increase in step frequency associated with the increase in spring stiffness. Therefore, an increase in hip flexion torque likely decreases the propulsive force and MoS, and this mechanism was explained by the intervening hip spring stiffness. Here, increased hip flexion torque and decreased the propulsive force and MoS are common characteristics in the elderly^2, 5, 10^, and a lower MoS in the elderly is also associated with their falling risk^10^. Therefore, for the elderly, increasing the hip quasi-stiffness during the swing phase may increase their falling risk. However, because the participants in this study were young adults, the relationship between hip quasi-stiffness and other variables in the elderly must be investigated before this mechanism can be applied to this population. Furthermore, the results of this study may be useful in the design of an unpowered hip exoskeleton^24, 25^ that uses the spring stiffness. This device, like the model in this study, exerts flexion torque during hip extension and extension torque during hip flexion due to the spring stiffness^24, 25^. However, studies have been limited to younger adults, and it is unclear whether this device can be adapted for rehabilitation of the elderly to prevent falls. The results of this study may be useful as a basis for examining the effects of spring stiffness adjustments on parameters related to their falling risk (step length, walking speed, propulsive force, and MoS).

## Conclusion

The powered simple walking model revealed the mechanism by which increased hip flexion torque decreases the propulsive force and MoS. A major part of this mechanism is the increase in step frequency associated with the increase in hip spring stiffness. The present results can reveal differences between the simple walking model and human gait, which may lead to a better understanding of human gait. Furthermore, our findings may help improve the control design of walking assistance devices, such as the unpowered hip exoskeleton, and also help us better understand the gait strategies of the elderly.

## Data Availability

The data which support the findings of this study are available from the corresponding author upon reasonable request.
